# Early introduction of solid foods and the low prevalence of food allergy and atopic eczema among preterm children during the first year of life

**DOI:** 10.1186/2045-7022-5-S3-O1

**Published:** 2015-03-30

**Authors:** Jaakko Yrjänä, Marita Valkama, Petri Kulmala

**Affiliations:** 1 Department of Pediatrics, Medical Research Center Oulu, Oulu University Hospital and University of Oulu, Oulu, Finland; 2 Institute of Diagnostics, Department of Medical Microbiology and Immunology, Medical Research Center Oulu and University of Oulu, Oulu, Finland

## Background

Clinical experience indicates preterm children having less food allergies (FA) and atopic dermatitis (AD) than full-term children. This is despite the fact that solid foods are usually started earlier in preterm infants to ensure the growth and development.

## Methods

We aimed to study early nutrition, FA and AD in a retrospective study. All the preterm infants (<37 gestational weeks) born and having been regularly followed up at the Department of Pediatrics, Oulu University Hospital during the years 2008-2012 are included. 383 preterm infants were identified from the hospital patient registry. Follow-up data from outpatient clinic visits until the age of 1 year was available in 31/157 (20%) of preterm (33-37 gestational weeks), 136/177 (77%) of very preterm (28-32 gestational weeks) and 42/49 (86%) of extremely preterm (<28 gestational weeks) infants, and these 209 children were included in the analyses. The frequencies of FA and AD were compared to our survey on infants from the general population.

## Results

The prevalence of FA and AD among 248 infants from the general population were 4.8% and 12.9%, respectively. In comparison, 4 (1.9%) of the all 209 preterm children had a confirmed diagnosis of FA (p=0.12) and 5 (2.4%) AD (p=0.0001). Among the very preterm infants only 1 (0.7%) had FA (p=0.038) and 4 (2.9%) had AD (p=0.001). No FA (p=0.23) or AD (p=0.007) were diagnosed among the extremely preterm infants by the age of 1 year. As calculated from the expected time of labor the mean age at the first introduction of fruits, vegetables, meat and grain to the diet in all preterm infants were 0.9, 1.0, 2.2 and 3.6 months, respectively. These mean ages among the very preterm babies were 0.5, 0.6, 1.9 and 3.2 months, and among the extremely preterm babies 0.02, 0.1, 1.3 and 2.8 months, respectively.

## Conclusion

Solid foods were introduced to the diet of preterm infants very early. However, the prevalence of FA and AD were low. These findings will be verified in a larger study population. Furthermore, a prospective study is planned to investigate relationship between early nutrition and prevalence of atopic disease in preterm infants.

